# Supplementary figures and images for: Phylogenomic Analyses Support Traditional Relationships within Cnidaria
Source: PLoS One. 2015 Oct 14;10(10):e0139068. doi: 10.1371/journal.pone.0139068 (PMC4605497; doi:10.1371/journal.pone.0139068)

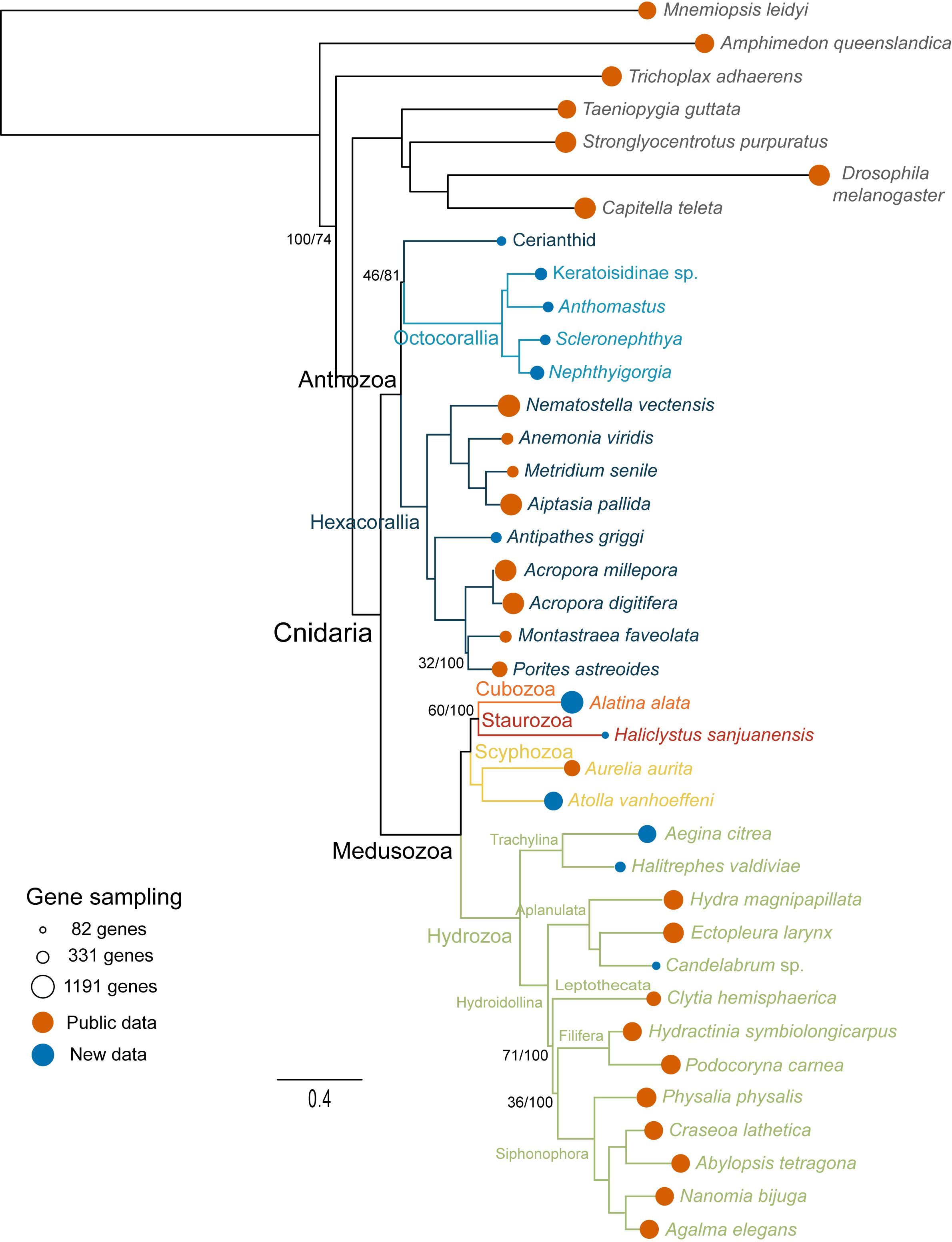

Supplement: S1 Fig — Branch support values correspond to percent ML-bootstrap values/percent Bayesian posterior probabilities. No values are shown for branches with 100/100 support. The areas of the lollipops, at the branch tips, are proportional to the number of genes sampled. (TIF) [file pone.0139068.s001.tif]

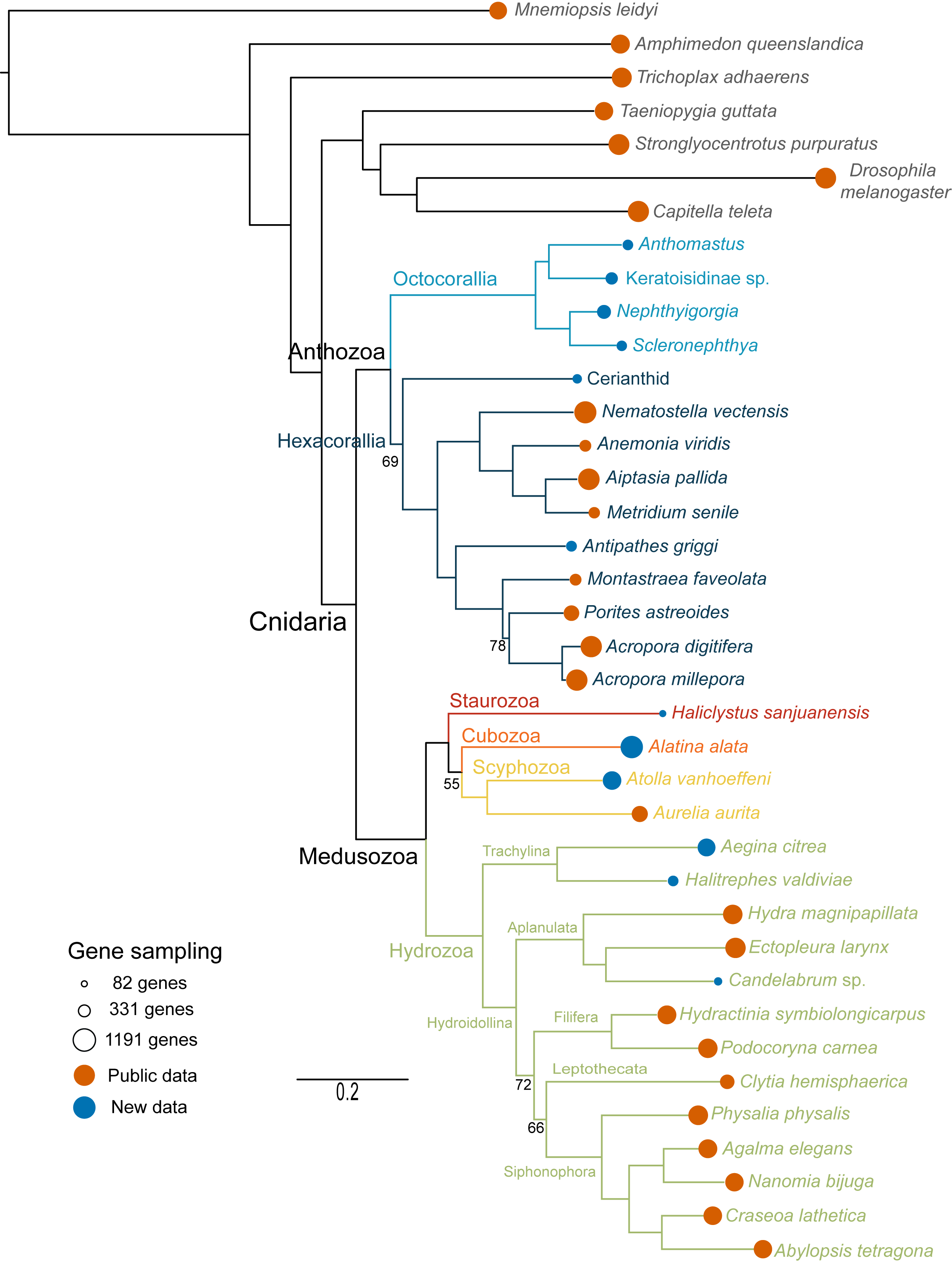

Supplement: S2 Fig — Branch support values correspond percent bootstraps. No values are shown for branches with 100% support. The areas of the lollipops, at the branch tips, are proportional to the number of genes sampled. (TIF) [file pone.0139068.s002.tif]

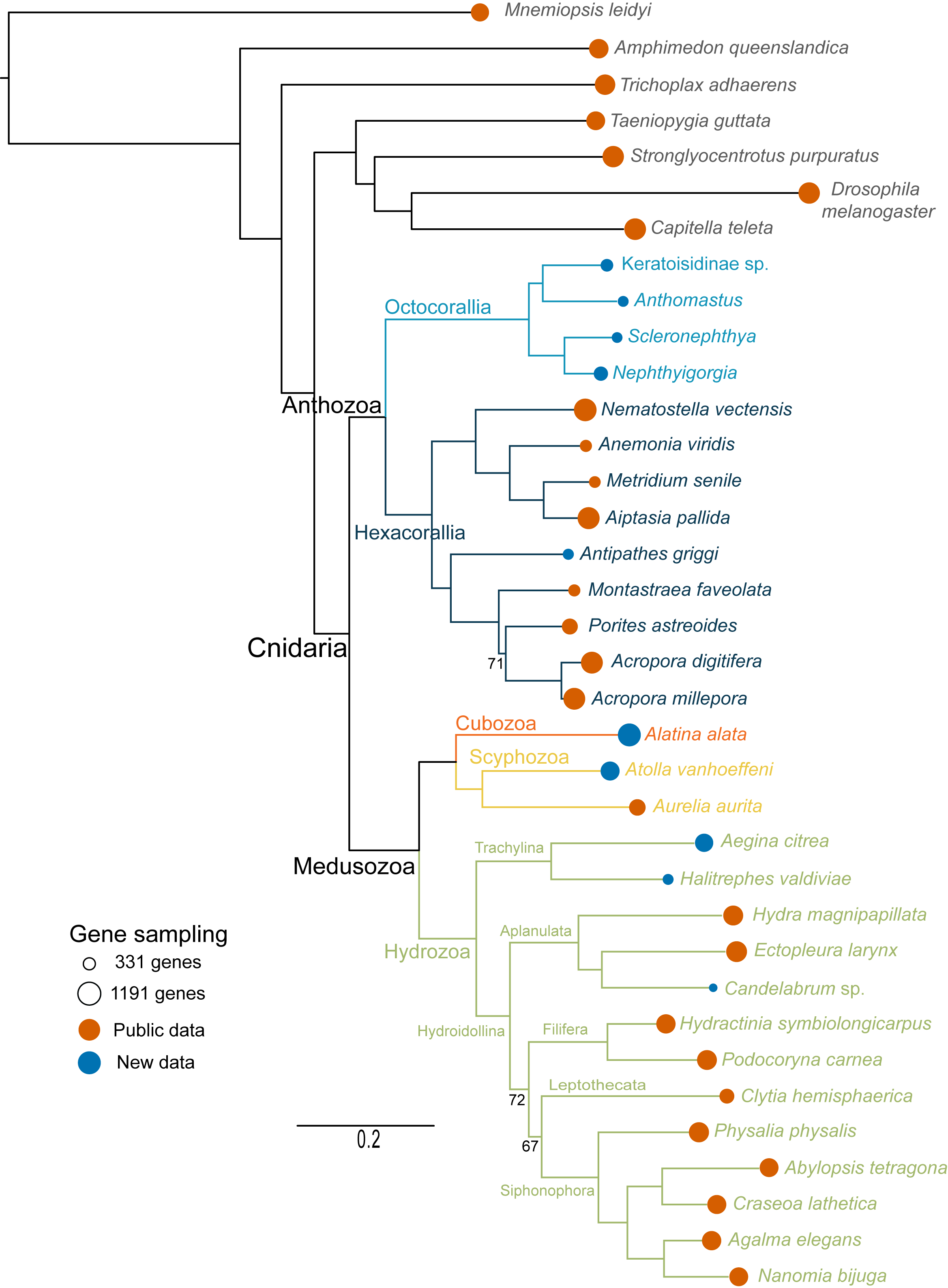

Supplement: S3 Fig — Branch support values correspond to percent bootstraps. No values are shown for branches with 100% support. The areas of the lollipops, at the branch tips, are proportional to the number of genes sampled. (TIF) [file pone.0139068.s003.tif]

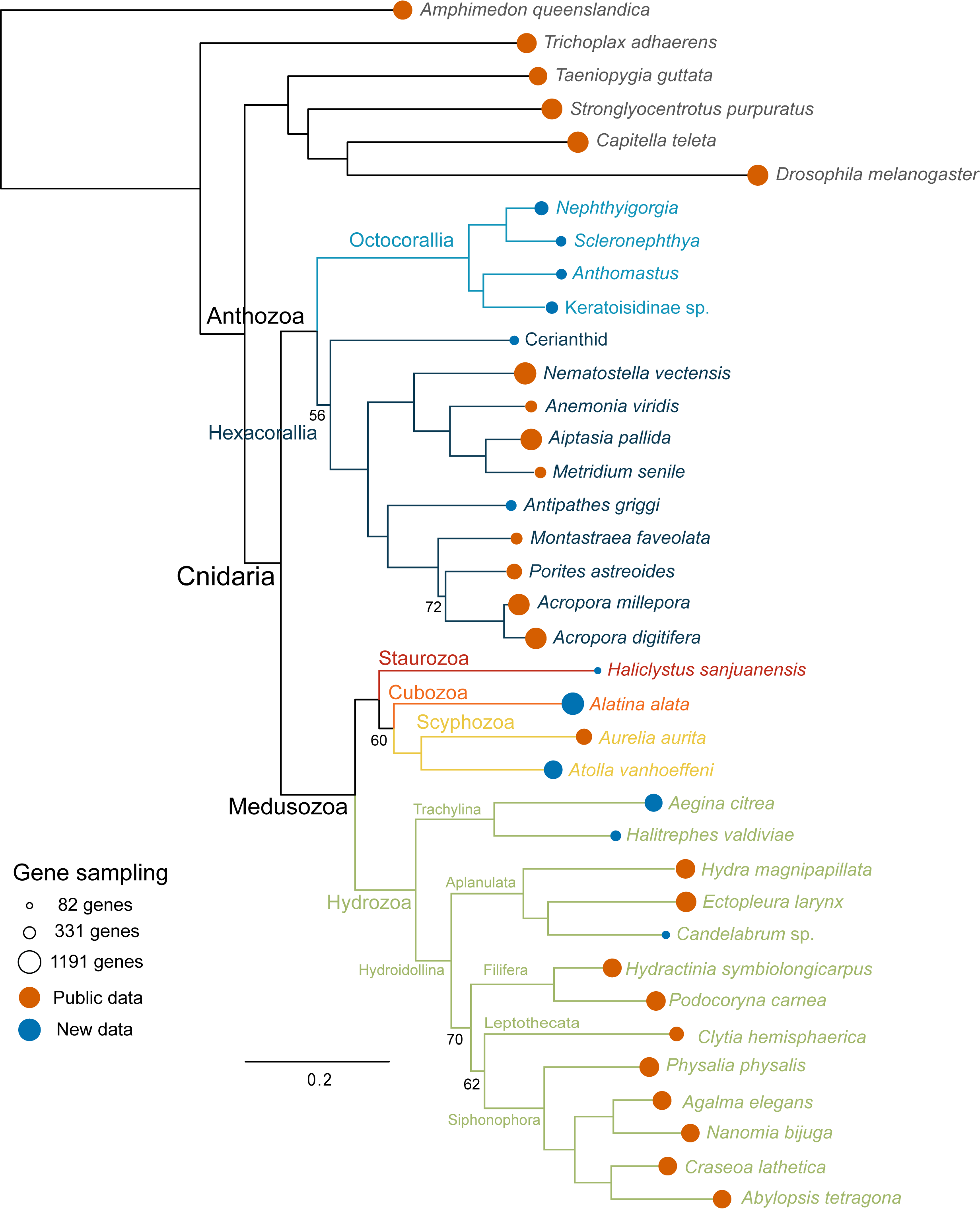

Supplement: S4 Fig — Branch support values correspond percent bootstraps. No values are shown for branches with 100% support. The areas of the lollipops, at the branch tips, are proportional to the number of genes sampled. (TIF) [file pone.0139068.s004.tif]

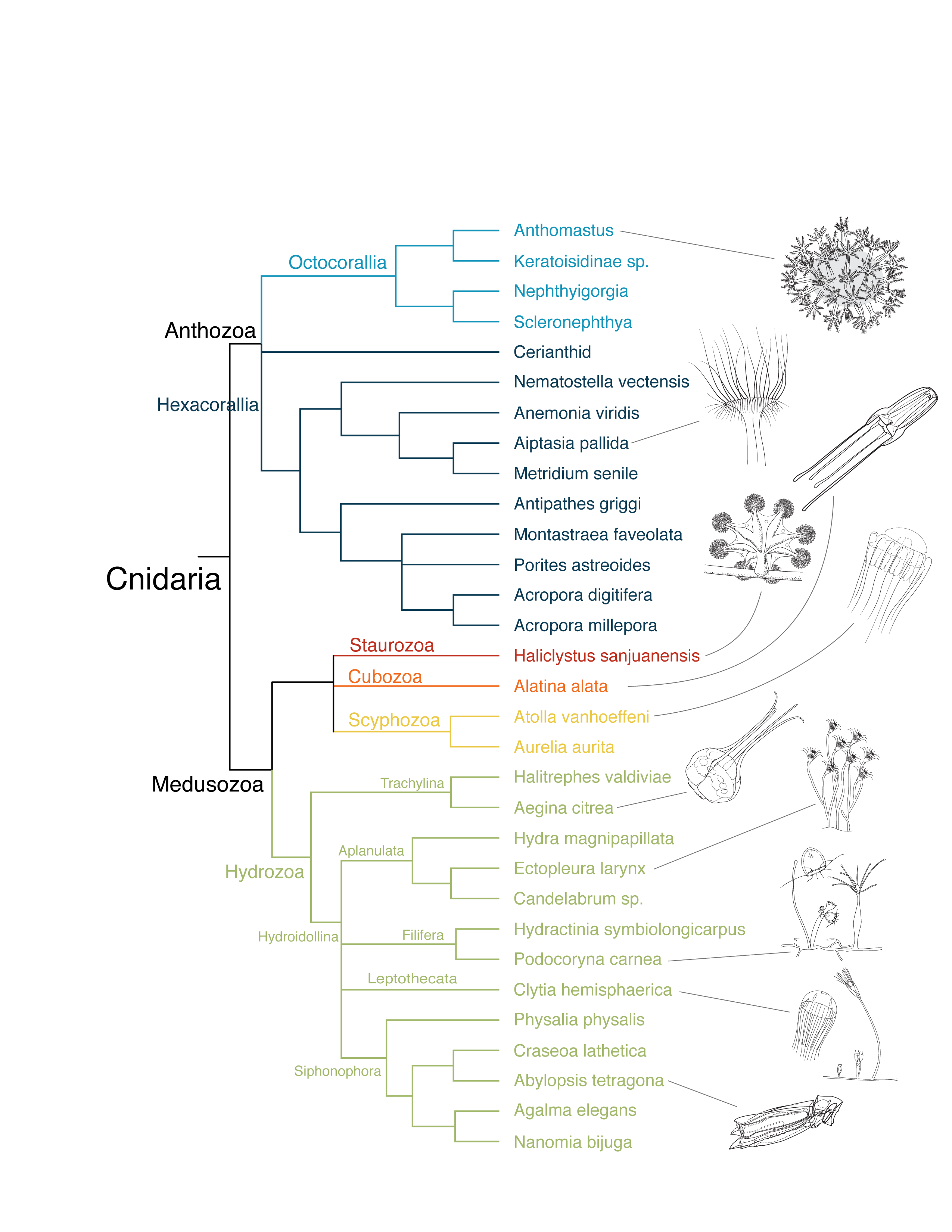

Supplement: S5 Fig — Cladogram of Cnidaria based on phylogeny in Fig 4. Branches that did not receive 100% support in ML and Bayesian analyses are collapsed to polytomies. (TIF) [file pone.0139068.s005.tif]
